# Evaluation of the clinical behavior of unclassified renal cell carcinoma and its imaging findings on computed tomography and magnetic resonance imaging based on World Health Organization (WHO) 2022

**DOI:** 10.1007/s11604-023-01484-1

**Published:** 2023-08-19

**Authors:** Akira Yamamoto, Tsutomu Tamada, Atsushi Higaki, Yuki Arita, Yoshiko Ueno, Takamichi Murakami, Masahiro Jinzaki

**Affiliations:** 1 Department of Radiology, Kawasaki Medical School, 577 Matsushima, Kurashiki, Okayama 701-0192 Japan; 2 Department of Radiology, Keio University School of Medicine, Tokyo, Japan; 3 Department of Radiology, Kobe University Graduate School of Medicine, Kobe, Japan

**Keywords:** Unclassified, Renal cell carcinoma, Clinical behavior, Imaging findings

## Abstract

**Objectives:**

To ascertain the clinical behaviors of unclassified renal cell carcinoma (RCC) and its characteristic imaging findings on CT and MRI.

**Methods:**

Subjects in this retrospective study were 10 patients who had received a histological diagnosis of unclassified RCC based on World Health Organization (WHO) 2022 and who had undergone CT and/or MRI prior to surgery. In terms of clinical behaviors, TNM classification, stage, postoperative recurrence, time to recurrence, and postoperative survival were evaluated. In terms of imaging findings, tumor size, growth pattern, CT density, dynamic contrast-enhancement (DCE) pattern, internal appearance, presence of a pseudocapsule, and signal intensity on MRI were evaluated. We compared clinical behaviors and imaging findings, and investigated associations between them.

**Results:**

One patient could not be followed-up due to death from other causes. Postoperative recurrence was observed in 4 patients, all of whom had Stage 3 RCC. In the remaining 5 patients without recurrence, all 5 patients showed Stage 2 or below. On imaging, unclassified RCC tended to be large (58.7 mm) and solid (100%), and heterogeneous interiors (80%), cystic degeneration (80%) and high intensity on diffusion-weighted imaging (DWI) (71.4%) were common. Comparing patients with and without recurrence, the following findings tended to differ between recurrence and recurrence-free groups: tumor size (73.4 ± 33.9 mm vs. 50.2 ± 33.9 mm, P = 0.286), growth pattern (invasive: 100% vs. 0%, expansive: 0% vs. 100%, P = 0.008 each), DCE pattern (progressive enhancement pattern, 66.7% vs. 0%, washout pattern, 0% vs. 66.7%, P = 0.135 each) and presence of a pseudocapsule (25% vs. 80%, P = 0.167).

**Conclusion:**

The clinical behavior of unclassified RCC varies widely. Although imaging findings are also variable, findings of large, heterogeneous tumors with cystic degeneration and high intensity on DWI were common. Several imaging findings such as large size, invasive growth, progressive enhancement pattern and no pseudocapsule may enable prediction of prognosis in unclassified RCC.

## Introduction

Renal cell carcinoma (RCC) is an important urological pathology. As a malignant tumor, RCC is classified into many subtypes that markedly impact the prognosis [1]. Unclassified RCC in World Health Organization (WHO) 2016 includes renal tumors that do not fit any of the identified histological subtypes [2, 3]. As a rare subtype, unclassified RCC represents 2–6% of renal epithelial tumors in adults [4]. Compared to the 14 subtypes in WHO 2016, WHO 2022 divides RCC pathologically into six groups and classifies 22 subtypes [5, 6]. Subtypes that were defined as unclassified RCC in WHO 2016 are now referred to as renal cell carcinoma, not otherwise specified (NOS) in WHO 2022. Subtypes showing characteristics similar to oncocytoma or chromophobe RCC without falling into either category are now classified as other oncocytic tumors of the kidney. Recent developments in pharmacotherapy for RCC have been dramatic, with a number of studies reporting extremely interesting findings not only for clear cell RCC, but also for non-clear cell RCC [7–9]. Current immunotherapeutic approaches for non-clear cell RCC also hold potential promising, with response rates over 30% being reported [10, 11]. Although many studies have reported that unclassified RCC includes a certain percentage of non-clear cell tumors, the lack of systematic central reviews and the wide histological spectrum mean that interpreting the results remains difficult and no standard therapies have been established [7–9]. This subtype includes relatively undifferentiated tumors and also includes tumors with a sarcomatous histology. For this reason, unclassified RCCs are treated as tumors with poor prognosis, and no specific clinical studies of unclassified RCC have been conducted under current treatment policies [8, 12]. However, some lesions do not exhibit an undifferentiated presentation and are not sarcomatous lesions despite not fitting into any of the existing histological subtypes, and these have yet to be characterized in detail despite the possibility that their responses to treatment may vary. Although a number of studies have investigated the clinical behaviors and prognoses and the variety of pathological findings for unclassified RCC [13], the basic conclusion was that imaging findings are diverse [14]. No study has yet addressed them in detail, nor has any published study reported the associations between a wide range of clinical behaviors and imaging findings. Our objective in this study was to ascertain the clinical behavior of unclassified RCC and its imaging findings on computed tomography (CT) and magnetic resonance imaging (MRI), and to investigate the associations between the diverse behaviors and imaging findings.

## Materials and methods

### Patients

This retrospective study was approved by the institutional review boards at each of the three participating hospitals, and the requirement for informed consent was waived. We reviewed pathology reports from the three institutions from June 2008 to June 2018, identifying a total of 11 patients diagnosed with unclassified RCC from postoperative histology conducted by experienced urological pathologists in these three institutions. The postoperative courses of these cases were retrospectively reviewed in July 2021. Furthermore, all cases were pathologically re-diagnosed in March 2023 in accordance with WHO 2022 criteria, and all cases were diagnosed as “RCC, NOS (unclassified RCC)”. One of the 11 patients was excluded from analysis because more than 1 year had passed between imaging investigations and surgery. The cases of remaining 10 patients (6 men, 4 women; mean age, 63.8 years; range, 44–82 years) were included for analysis. All 10 patients had undergone preoperative CT. Abdominal CT images were obtained using a multidetector-row CT unit as unenhanced CT in 3 cases, contrast-enhanced CT only in 1 case, and unenhanced and contrast-enhanced CT in 6 cases (single-phase contrast in 1 case, double-phase contrast in 1 case, and triple-phase contrast in 4 cases). The single-phase contrast CT was performed to enhance the nephrographic phase, the double-phase to enhance the corticomedullary and excretory phases, and the triple-phase to enhance corticomedullary, nephrographic, and excretory phases. Eight patients had undergone MRI. Abdominal MRI was performed using a 1.5- or 3.0-T MRI unit. T1-weighted imaging (7/8 cases), T2-weighted imaging (8/8 cases), and diffusion-weighted imaging (DWI) (b = 0 and 800 or 1000 s/mm^2^) (7/8 cases) were performed, and contrast enhancement was applied in 5 cases (single-phase contrast in 1 case, triple-phase contrast in 4 cases). Single-phase contrast MRI was scanned to enhance the corticomedullary phase, and the triple phase to enhance the corticomedullary, nephrographic, and excretory phases. The mean interval between imaging investigations and surgery was 54.3 days (range, 0–102 days) for CT and 44.1 days (range, 1–113 days) for MRI. Table 1 shows the attributes of the 10 study subjects, in order of the date of surgery.

**Table 1:** Patient information for the 10 subjects in this study

Patient	Age	Sex	CT	MRI	Time from CT to surgery (days)	Time from MRI to surgery (days)
1	53	M	Unenhanced-CT with CE-CT (2 phase)	MRI with CE (3 phase)	6	5
2	72	M	Unenhanced-CT	MRI with no contrast	36	27
3	48	M	Unenhanced-CT	MRI with no contrast	89	30
4	58	F	Unenhanced-CT with CE-CT (3 phase)	None	102	N/A
5	80	F	CE-CT (1 phase)	None	93	N/A
6	72	M	Unenhanced-CT with CE-CT (3 phase)	MRI with CE (3 phase)	91	105
7	82	F	Unenhanced-CT with CE-CT (3 phase)	MRI with CE (3 phase)	34	34
8	72	F	Unenhanced-CT	MRI with CE (3 phase)	0	113
9	57	M	Unenhanced-CT with CE-CT (1 phase)	MRI with no contrast (only T2WI)	62	38
10	44	M	Unenhanced-CT with CE-CT (3 phase)	MRI with CE (1 phase)	30	1

*CE* Contrast enhanced

### Clinical analysis

Clinical data were obtained from the retrospective review of medical records conducted in July 2021. We analyzed postoperative TNM classification, stage, surgical procedure, Fuhrman grade obtained by postoperative pathological diagnosis, postoperative recurrence, time to recurrence if recurrence occurred, recurrence-free time if recurrence did not occur, mortality, cause of death if death occurred, and postoperative survival.

### Imaging analysis

In terms of imaging findings, we evaluated the following: tumor size; whether the lesion was solid or cystic; growth pattern; unenhanced CT density; dynamic contrast enhancement (DCE) pattern; internal appearance of the tumor; presence or absence of an internal cystic component, fat component, intratumoral hemorrhage or pseudocapsule; DWI signal intensity (b = 800 or 1000 s/mm^2^); apparent diffusion coefficient (ADC); and signal intensity on T1- and T2-weighted imaging using the methods described below, on the basis of agreement between two diagnostic radiologists with 16 and 24 years of experience. Tumor size and growth pattern were measured and evaluated on images from CT or MRI, choosing the modality in which the lesion was most clearly demarcated. Classification as a solid or cystic lesion was conducted by observing the entire lesion on contrast-enhanced or T2-weighted images. Unenhanced CT density was assessed visually on a three-grade scale as hyperdense, isodense, or hypodense compared with healthy renal cortex. DCE pattern was only evaluated in patients who had undergone both unenhanced scanning and dynamic triple-phase contrast enhancement on CT or MRI (corticomedullary phase, nephrographic phase, and excretory phase). Contrast enhancement ratios were calculated [(post-contrast value—pre-contrast value)/pre-contrast value], time-density curves were produced and the pattern was classified into one of the following three patterns: progressive (gradually increasing contrast effect); plateau (early enhancement with persistent contrast effect); or washout (early enhancement followed by washout). Internal appearance of the tumor was classified visually as homogeneous or heterogeneous on post-contrast CT and/or T2-weighted imaging. The presence or absence of a cystic component was evaluated on contrast investigations and/or T2-weighted imaging. The presence of fat was defined as a decrease in signal in the opposed-phase compared to the in-phase on chemical shift imaging. The presence or absence of intra-tumoral hemorrhage was evaluated in terms of the presence or absence of a signal decrease in the in-phase reflecting hemosiderin on chemical shift imaging. The presence or absence of a pseudocapsule was evaluated during the contrast excretory phase on CT or MRI and/or T2-weighted imaging. Signal intensity on T1-weighted imaging, T2-weighted imaging, and DWI were assessed visually using a three-grade scale of hyperintense, isointense, or hypointense compared with healthy renal cortex. The investigators were blinded to pathological information and any clinical information.

We also compared clinical behaviors and imaging findings, and investigated the associations between them.

### Statistical analysis

Clinical, MRI and CT indices were compared between recurrence and recurrence-free groups using Fisher’s exact test, the χ^2^ test, and the Mann–Whitney U-test. Tumor size and tumor ADC were compared using the Mann–Whitney U-test. The frequencies of tumor appearances (solid or cystic), growth patterns (expansive or invasive), unenhanced CT density, signal intensity on T1WI and T2WI (high, iso, or low), DCE pattern (progressive, plateau, or washout), internal appearance (homogeneous or heterogeneous), fat component and pseudocapsule (presence or absence) were compared using Fisher’s exact test or the χ^2^ test. Inter-reader agreement was calculated using Cohen’s kappa statistic. When Kappa cannot be computed because variable is a constant, the raw agreement (number of cases in agreement/total number of cases) is adapted instead of the Kappa value. Data were analyzed using SPSS for Windows version 24.0 software (SPSS Inc., Chicago, IL, U.S.). Values of P < 0.05 were indicated significance.

## Results

### Clinical behaviors

Table 2 shows the detailed pre- and postoperative statuses of all 10 patients, in order of the date of surgery. Preoperatively, RCC was Stage 1 in 4 patients, Stage 2 in 2, and Stage 3 in 4. The surgical procedure was total nephrectomy in 7 cases and partial nephrectomy in 3. Pathological Fuhrman grade was G1 in 2 cases, G2 in 1, G3 in 5, G4 in 1, and indeterminate in 1. Four patients developed recurrence during follow-up and the mean time to postoperative recurrence was 224.5 days (range, 129–358 days). Three patients died during postoperative follow-up; 2 deaths were due to cancer, occurring on postoperative days 129 and 358. One patient died of sepsis after abdominal aneurysm surgery on postoperative day 129. Six patients remained recurrence-free postoperatively. Excluding the patient who died of sepsis and could not be followed-up long term, mean duration of follow-up in the other 5 recurrence-free patients was 3148.7 days (range, 1122–3642 days). The 10 patients with unclassified RCC exhibited a broad range of clinical behaviors, from cancer death on postoperative day 129 to up to 10 years of recurrence-free survival.

**Table 2:** Detailed information of pre- and post-operative patient status of all 10 patients

Patient	TNM classification	Stage	Surgical procedure	Fuhrman grade	Recurrence	Time to recurrence (days)	Recurrence-free follow-up (days)	Mortality	Cause of death	Survival time (days)
1	pT3a. N1, M0	Stage 3b	Total	G3	Yes	129	N/A	Died	Cancer	129
2	pT3a, N1, M0	Stage 3b	Total	G4	Yes	358	N/A	Died	Cancer	358
3	pT1b, N0, M0	Stage 1b	Total	G3	No	N/A	129	Died	Sepsis	129
4	pT1b, N0, M0	Stage 1b	Partial	G2	No	N/A	1122	Surviving	N/A	N/A
5	pT1a, N0, M0	Stage 1a	Partial	G1	No	N/A	2996	Surviving	N/A	N/A
6	pT1a, N0, M0	Stage 1a	Partial	G1	No	N/A	2462	Surviving	N/A	N/A
7	pT3, N1, M0	Stage 3a	Total	None	Yes	329	N/A	Surviving	N/A	N/A
8	pT2, N0, M0	Stage 2b	Total	G3	No	N/A	3642	Surviving	N/A	N/A
9	pT2, N0, M0	Stage 2a	Total	G3	No	N/A	3342	Surviving	N/A	N/A
10	pT3a, N0, M0	Stage 3a	Total	G3	Yes	120	N/A	Surviving	N/A	N/A

*Total* Total nephrectomy

*Partial* Partial nephrectomy

In all 4 patients who developed postoperative recurrence, RCC was Stage 3 preoperatively, and two of these patients died within 1 year postoperatively, exhibiting a highly malignant course. Fuhrman grade also tended to be high (G3 in 2 cases and G4 in 1). The 6 patients who were postoperatively recurrence-free were preoperative Stage 2 or below (Stage 1 in 4 cases, Stage 2 in 2 cases), and excluding the patient who died of sepsis and could not be followed up long term, none of the other 5 patients developed recurrence during the minimum of 3 years of follow-up (mean, 2709.2 days; range, 1104–3642 days), exhibiting a tendency toward lower clinical malignancy. Fuhrman grade also tended to be low (G1, n = 2; G2, n = 1; G3, n = 2).

### Image findings on CT and MRI

**Fig. 1:**
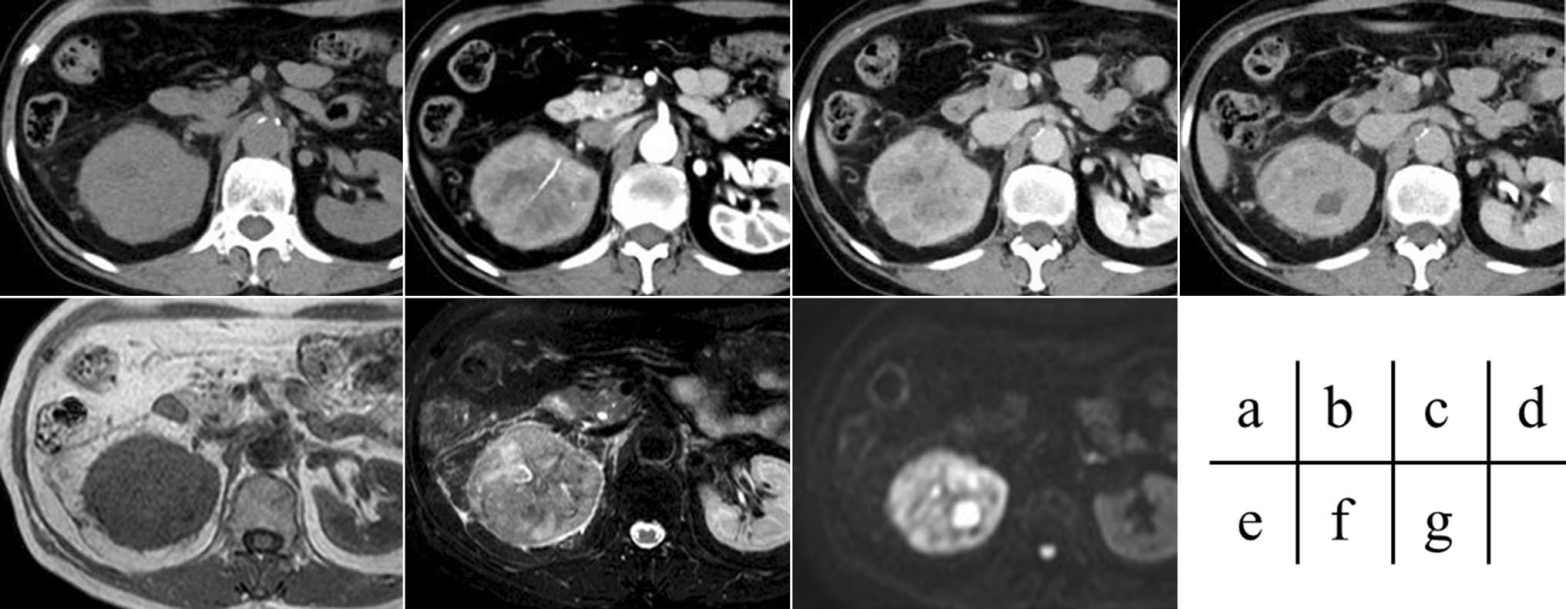
Images from an 82-year-old woman with unclassified RCC. A large mass lesion with invasive growth is apparent in the right kidney. The tumor is isodense on unenhanced CT (**a**) and exhibits a progressive pattern on DCE imaging (**b**–**d**). The tumor appears isointense on T1-weighted imaging (**e**), hypo- to hyperintense with heterogeneous internal appearance on T2-weighted imaging (**f**) and hyperintense on DWI (**g**), with an evident cystic component. Unclassified RCC was diagnosed after surgery. Recurrence was confirmed 329 days after surgery

**Fig. 2:**
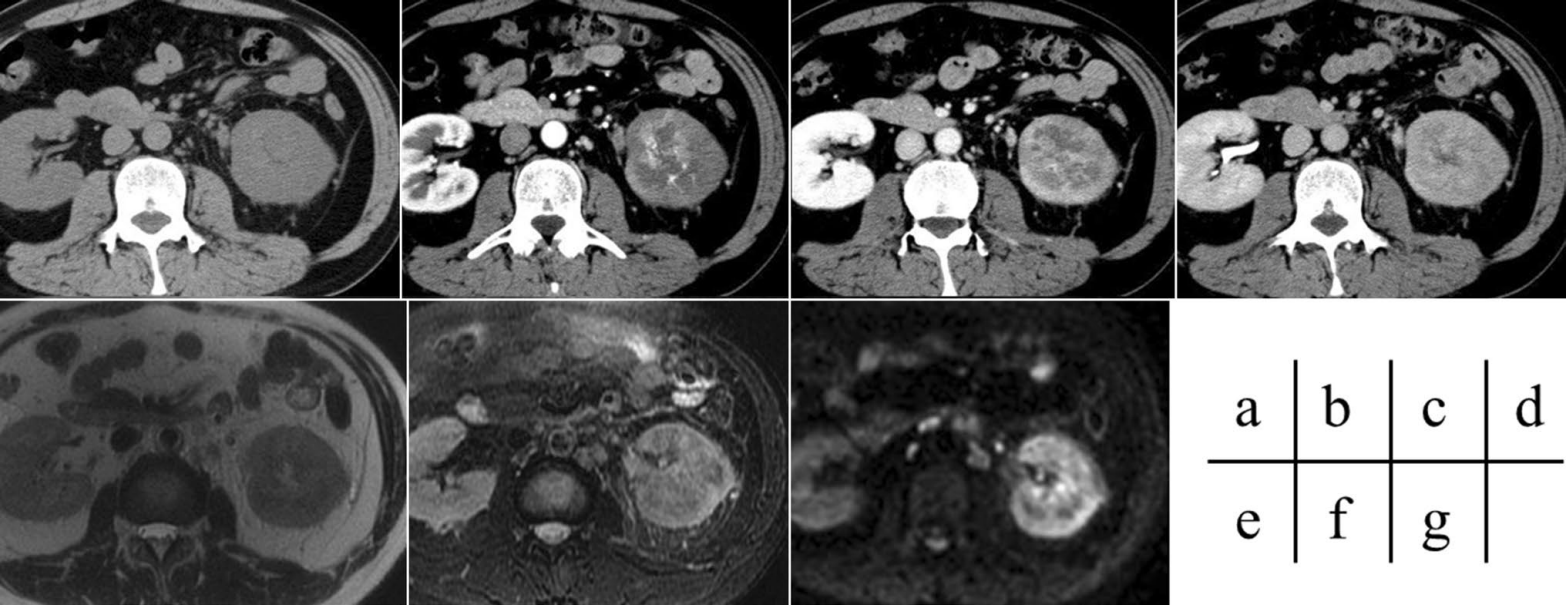
Images from a 44-year-old man with unclassified RCC. A mass lesion with invasive growth is apparent in the left kidney. The tumor appears isointense on unenhanced CT (**a**) and exhibits a progressive pattern on DCE imaging (**b**–**d**). The tumor also appears isointense on T1-weighted imaging (**e**), mildly hypointense on T2-weighted imaging (**f**), and hyperintense on DWI (**g**). No pseudocapsule is clearly identifiable. Unclassified RCC was diagnosed after surgery. The patient developed postoperative recurrence and died of cancer on Day 358

**Fig. 3:**
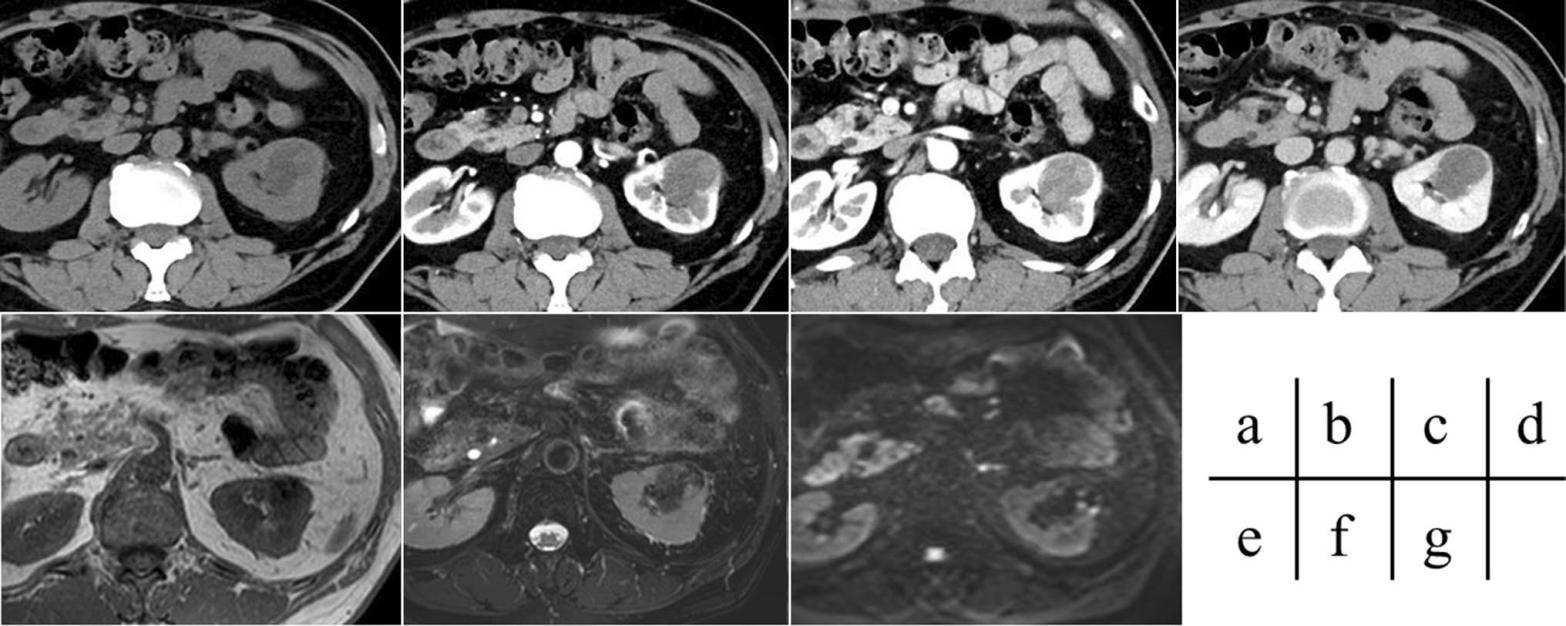
Images from a 72-year-old man with unclassified RCC. A mass lesion with expansive growth is identified in the left kidney. The tumor appears hypodense on unenhanced CT (**a**) and exhibits a mild washout pattern on DCE imaging (**b**–**d**). The tumor appears mildly hypointense on T1-weighted imaging (**e**) and hypointense on T2-weighted imaging (**f**) with suspected hemorrhage and hypointense on DWI (**g**). A partial pseudocapsule is identified. Unclassified RCC was diagnosed after surgery. As of postoperative Day 2462, the patient remains free from recurrence

In terms of imaging findings, mean tumor size was 58.7 mm (range, 18.8–119.4 mm). The tumor was solid in all 10 cases, with no cystic tumor. The growth pattern was expansive in 6 of 10 cases (60%) and invasive in 4 of 10 (40%). Unenhanced CT density was hyperdense in 2 of 9 cases (22.2%), isodense in 3 of 9 cases (33.3%), and hypodense in 4 of 9 cases (44.4%). The DCE pattern was progressive in 2 of 6 lesions (33.3%), plateau in 2 of 6 cases (33.3%), and washout in 2 of 6 cases (33.3%). Internal appearance of the tumor was homogeneous in 2 of 10 lesions (20%) and heterogeneous in 8 of 10 cases (80%). An intralesional cystic component was present in 8 of 10 lesions (80%), and an intralesional fat component was seen in 1 of 6 lesions (16.7%). Intratumoral hemorrhage was evident in 2 of 6 lesions (33.3%). A partial pseudocapsule was seen in 5 of 10 cases (50%) and no pseudocapsule at all in 5 of 10 cases (50%). On DWI, 5 of 7 lesions (71.4%) were hyperintense, 0 of 7 lesions (0%) were isointense, and 2 of 7 lesions (28.6%) were hypointense. The ADC was measurable in 4 lesions, with a mean value of 1.36 ± 0.64 × 10^–3^ mm^2^/s (range, 0.84–2.29 × 10^–3^ mm^2^/s). Signal intensity on T1-weighted imaging was hyperintense in 0 of 7 lesions (0%), isointense in 3 of 7 lesions (42.9%), and hypointense in 4 of 7 lesions (57.1%). Signal intensity on T2-weighted imaging was hyperintense in 2 of 8 lesions (25%), isointense in 1 of 8 lesions (12.5%), and hypointense in 5 of 8 lesions (62.5%). The characteristics of those imaging findings seen comparatively frequently for unclassified RCC were comparatively large size (mean, 58.7 ± 32.5 mm), solid tumors (100%) that were internally heterogeneous (80%), with a cystic component (80%) but no fat component (16.7%), and appearing hyperintense on DWI (71.4%) (Fig. 1). Table 3 shows the imaging findings of all 10 patients (Figs. 2, 3).

**Table3:** Tumor characteristics on image findings of all 10 patients

Patient	Tumor size (mm)	Cystic or solid lesion	Growth pattern	Unenhanced-CT density	DCE pattern	Tumor internal appearance	Cystic component	Fat component	Intra-tumoral hemorrhage	Pseudo capsule	DWI	ADC value (× 10^−3^mm^2^/s)	T1WI	T2WI
1	37.6	Solid	Invasive	High	Progressive	Homogeneous	No	No	No	No	High		Low	Low
2	119.4	Solid	Invasive	High		Heterogeneous	Yes	No	Yes	No	High	1.07	Iso	Low
3	43.3	Solid	Expansive	Low		Heterogeneous	Yes	No	No	Yes	Low	2.29	Low	High
4	43.0	Solid	Expansive	Low	Washout	Heterogeneous	No			No				
5	18.8	Solid	Expansive			Heterogeneous	Yes			Yes				
6	31.4	Solid	Expansive	Low	Washout	Heterogeneous	Yes	No	Yes	Yes	Low	1.25	Iso	Low
7	69.1	Solid	Invasive	Iso	Plateau	Heterogeneous	No	Yes	No	No	High	0.84	Iso	Low
8	106.8	Solid	Expansive	Low	Plateau	Heterogeneous	Yes	No	No	Yes	High		Low	Low
9	70.1	Solid	Expansive	Iso		Heterogeneous	Yes			Yes				High
10	67.5	Solid	Invasive	Iso	Progressive	Heterogeneous	Yes			Yes	High		Low	Iso
All cases	Mean ± SD 58.7 ± 32.5	Solid 10/10(100%) Cystic 0/10 (0%)	Expansive 6/10 (60%) Invasive 4/10 (40%)	High 2/9 (22.2%) Iso 3/9 (33.3%) Low 4/9 (44.4%)	Progressive 2/6 (33.3%) Plateau 2/6 (33.3%) Washout 2/6 (33.3%)	Homogeneous 2/10 (20%) Heterogeneous 8/10 (80%)	Yes 8/10 (80%) No 2/10 (20%)	Yes 1/6 (16.7%) No 5/6 (83.3%)	Yes 2/6 (33.3%) No 4/6(66.7%)	Yes 5/10 (50%) No 5/10(50%)	High 5/7 (71.4%) Iso 0/7 (0%) Low 2/7 (28.6%)	Mean ± SD 1.36 ± 0.64 (n = 4)	High 0/7 (0%) Iso 3/7 (42.9%) Low 4/7 (57.1%)	High 2/8 (25%) Iso 1/8 (12.5%) Low 5/8 (62.5%)

### Relationships between clinical behavior and imaging findings

Of the 4 patients who developed postoperative recurrence, 2 (50%) died within 1 year. Fuhrman grade in those patients tended to be high, suggesting high malignancy. In contrast, after excluding the recurrence-free patient who died of sepsis and could not be followed-up long term, the other 5 recurrence-free patients remained recurrence-free for at least 3 years of follow-up and Fuhrman grade tended to be low, suggesting low malignancy.

We compared imaging findings between the 4 patients who developed postoperative recurrence (recurrence group) and the 5 patients who remained recurrence-free and could be followed-up long term (recurrence-free group). Table 4 shows the frequencies of image findings in the recurrence and recurrence-free groups. Image findings that tended to differ between recurrence and recurrence-free groups comprised tumor size (73.4 ± 33.9 mm vs. 50.2 ± 33.9 mm, respectively; P = 0.286), growth pattern (invasive: 100% vs. 0%, respectively; expansive: 0% vs. 100%, respectively; P = 0.008), DCE pattern (progressive enhancement pattern, 66.7% vs. 0%; washout pattern, 0% vs. 66.7%, respectively; P = 0.135) and presence of a pseudocapsule (25% vs. 80%, respectively; P = 0.167). These results suggest that large masses with no pseudocapsule that are growing invasively, appear iso- to hyperdense on unenhanced CT, and exhibit a progressive pattern on DCE imaging are more likely to be clinically highly malignant, whereas masses with a pseudocapsule that are growing expansively, appear hypodense on unenhanced CT, and exhibit a washout pattern on DCE imaging are more likely to be clinically comparatively less malignant.

**Table 4:** Comparison of image findings between recurrence group and recurrence-free group

Imaging feature		Recurrence group (n = 4)	Recurrence-free group (n = 5)	K values *raw agreement	P values
Tumor size (mm)	Mean ± SD	73.4 ± 33.9	50.2 ± 33.9		0.286
Solid or cystic tumor	Solid Cystic	4/4 (100%) 0/4 (0%)	5/5 (100%) 0/5 (0%)	0.889*	1.000
Growth pattern	Expansive Invasive	0/4 (0%) 4/4 (100%)	5/5 (100%) 0/5 (0%)	1.000	0.008
Unenhanced-CT density	High	2/4 (50%)	0/4 (0%)	0.610	0.069
	Iso	2/4 (50%)	1/4 (25%)		
	Low	0/4 (0%)	3/4 (75%)		
DCE pattern	Progressive	2/3 (66.7%)	0/3 (0%)	0.706	0.135
	Plateau	1/3 (33.3%)	1/3 (33.3%)		
	Washout	0/3 (0%)	2/3 (66.7%)		
Tumor internal appearance	Homogeneous	1/4 (25%)	1/5 (20%)	0.588	0.722
	Heterogeneous	3/4 (75%)	4/5 (80%)		
Cystic component	Yes No	3/4 (75%) 1/4 (25%)	4/5 (80%) 1/5 (20%)	0.696	0.722
Fat component	Yes No	1/3 (33.3%) 2/3 (66.7%)	0/2 (0%) 2/2 (100%)	1.000	0.600
Intra-tumoral hemorrhage	Yes No	1/3 (33.3%) 2/3 (66.7%)	1/2 (50%) 1/2 (50%)	0.667	0.700
Pseudo capsule	Yes No	1/4 (25%) 3/4 (75%)	4/5 (80%) 1/5 (20%)	0.750	0.167
Signal intensity on DWI	High	4/4 (100%)	1/2 (50%)	1.000	0.333
	Iso	0/4 (0%)	0/2 (0%)		
	Low	0/4 (0%)	1/2 (50%)		
ADC (× 10^−3^mm^2^/s)	Mean ± SD	0.96 ± 0.16 (n = 2)	1.25 (n = 1)		1.000
Signal intensity on T1WI	High	0/4 (0%)	0/2 (0%)	0.714	0.800
	Iso	2/4 (50%)	1/2 (50%)		
	Low	2/4 (50%)	1/2 (50%)		
Signal intensity on T2WI	High	0/4 (0%)	1/3 (33.3%)	0.731	0.714
	Iso	1/4 (25%)	0/3 (0%)		
	Low	3/4 (75%)	2/3 (66.7%)		

## Discussion

Because lesions that do not fit into any of the other histological subtypes are classed as “unclassified RCC,” both undifferentiated RCCs with few histological characteristics and RCCs exhibiting sarcomatous changes tend to be categorized under this subtype [8, 12]. Lesions in this category therefore tend to be highly malignant. However, other lesions with a somewhat differentiated histological presentation that are not histologically highly malignant are also categorized under this subtype if they lack the characteristics of other subtypes. The clinical behaviors and imaging findings may therefore be expected to vary substantially. Despite the small number of cases in this study, we found that in terms of clinical behavior, some patients died of early recurrence within 1 year postoperatively, whereas others survived recurrence-free for approximately 10 years after surgery. This difference was probably affected by the preoperative stage, but even after taking this into account, major differences in prognosis seem to remain and lesions with high histological malignancy may have already progressed to a more advanced clinical stage by the time of discovery. This is backed up by the fact that some difference in Fuhrman grade was also evident. Our imaging findings were also as diverse as previously reported [14]. However, we were able to identify a number of imaging findings on CT and MRI that were comparatively common in unclassified RCC. Unclassified RCC accounts for only 2–6% of RCCs, but should be included among the differential diagnoses for lesions that exhibit the findings on imaging investigations identified in this study; namely, comparatively large, solid, heterogeneous tumors with a cystic component but no fat component, appearing hyperintense on DWI.

The present results also suggest that this diverse clinical behavior can be predicted to some extent from imaging findings. Large tumors with an invasive growth pattern that appeared iso- to hyperdense on unenhanced CT, exhibited progressive enhancement pattern on DCE imaging, and did not have a pseudocapsule tended to be more malignant. Tumors with an expansive growth pattern that were hypodense on unenhanced CT, exhibited a washout pattern on DCE imaging, and had a pseudocapsule were likely to have a comparatively good prognosis despite being histologically categorized as unclassified RCC. Particularly in terms of growth patterns, although the number of cases was small, a significant difference was observed (P = 0.008). In a previous study that categorized unclassified RCCs into different pathological patterns and compared the results for those patterns with staging, tumors with a clear cell RCC-like phenotype or an oncocytoma/chromophobe RCC-like phenotype were identified at a significantly earlier stage than those with a collecting duct/papillary RCC-like/pure sarcomatoid phenotype [13]. In the present results, the characteristics of imaging findings in postoperative recurrence-free cases resembled those of clear cell RCC [15], and imaging findings may have reflected the pathological pattern. In clinical terms, this may represent useful information for defining postoperative follow-up intervals and periods.

This study showed a number of limitations. The first was the small sample size of only 10 patients. Although further studies including larger cohorts are required, the diverse clinical behaviors and imaging findings seen in this study were consistent with findings from previous reports and we were able to identify trends in the imaging findings of unclassified RCC and trends in imaging findings depending on malignancy. Second, because this was a multicenter study, the types of imaging devices used and imaging conditions were not standardized. However, the small number of patients pathologically diagnosed with unclassified RCC meant that a multicenter study was required to include a suitable number of study subjects. We did not consider that these differences exerted any major effect on the present results. Finally, diagnoses were made by different pathologists, but as each was made by a board-certified urological pathologist employed by the institution concerned, we consider that the results were all reliable and that this difference had no effect on the study findings.

In conclusion, we found that the clinical behaviors and imaging findings of unclassified RCC were diverse, as previously reported. However, we were able to identify trends in imaging findings that should form a basis for suspecting unclassified RCC. The imaging findings identified may help predict postoperative recurrence and prognosis due to the diverse clinical behaviors of unclassified RCC.
